# A laboratory test of evolutionary aging theories

**DOI:** 10.18632/aging.101215

**Published:** 2017-03-21

**Authors:** Tatiana Iouk, Vladimir I. Titorenko

**Affiliations:** Department of Biology, Concordia University, Montreal, Quebec H4B 1R6, Canada

**Keywords:** yeast, aging, natural aging-delaying, compounds, hormesis, evolution, ecosystems

A high‐throughput chemical genetic screen of chemical compounds from several commercial libraries has revealed that lithocholic bile acid (LCA), and some other bile acids, can slow yeast chronological aging [1]. The robust geroprotective effect of exogenously added LCA is due to its ability to enter chronologically aging yeast cells, be sorted to both mitochondrial membranes and alter mitochondrial lipidome [2]. This elicits considerable changes in mitochondrial morphology and functionality, thus allowing mitochondria to operate as a signaling platform that institutes and maintains an aging-delaying pattern of the entire cell [3].

LCA and other bile acids are mildly toxic molecules that cause a so-called ″hormetic″ stress response in animals; because bile acids elicit chemical hormesis, they act as endobiotic geroprotective regulators that can delay the onset and slow the progression of animal aging [4]. Yeast cells do not synthesize LCA and other bile acids found in animals [5]. To explain how these natural molecules can delay yeast chronological aging, we proposed a hypothesis of the hormetic selective forces driving the evolution of longevity regulation mechanisms within ecosystems [5]. This hypothesis posits that after animals inhabiting an ecosystem release bile acids into the environment, these mildly toxic chemicals may create hormetic selective force that drives the evolution of certain protective mechanisms in yeast within this ecosystem. These mechanisms protect yeast against bile acid-induced cellular damage [5]. Our hypothesis further suggests that some of these mechanisms of protection against broad cellular damage elicited by bile acids can also protect yeast against damage and stress accumulated purely with age. Therefore, those yeast species that have developed such longevity regulation mechanisms are expected to live longer [5]. As a laboratory test of this hypothesis, we recently conducted a multistep selection of long-lived yeast species by a lasting exposure of yeast cells to different concentrations of exogenously added LCA [6]. This test yielded twenty long-lived yeast mutants, three of which were capable of maintaining their considerably prolonged chronological lifespans after numerous passages in medium without LCA [6]. Our genetic analyses have revealed that the extended longevity of each of the three selected long-lived yeast mutants was a polygenic genetic trait caused by mutations in more than two nuclear genes [6]. In further support of the hypothesis on hormetic selective forces driving the ecosystemic evolution of longevity regulation mechanisms, none of the yeast cells that were not exposed to exogenous bile acids had chronological lifespan above a species-specific age [6]. Thus, unlike yeast cells exposed to exogenous LCA, yeast cells that were not subjected to such exposure were unable to develop mechanisms of protection against age-related damage and to live longer [6].

We then used the selected long-lived yeast mutants for a laboratory test of evolutionary theories of programmed or non-programmed aging [7]. Programmed aging theories assume that all organisms have evolved certain active mechanisms for limiting their lifespans at a species-specific age, whereas non-programmed aging theories postulate that such mechanisms cannot exist because organismal lifespan is limited at a species-specific age due to the lack of any evolutionary force [7]. In support of programmed aging theories, we found that the dominant polygenic trait increasing the chronological lifespan of each of the three selected long-lived mutants 1) does not alter the key features of early-life fitness, including the exponential growth rate, efficiency of post-exponential growth and fecundity; 2) enhances other key features of early-life fitness by increasing cell resistance to chronic exogenous stresses and by decreasing cell susceptibility to exogenously induced modes of programmed death; and 3) lowers the relative fitness of the mutant strain in direct competition with the parental wild-type strain exhibiting shorter lifespan, thus being forced out of the ecosystem by the strain whose lifespan is limited at a species-specific age [7].

In sum, a laboratory test of evolutionary aging theories provided evidence that yeast cells have evolved some active mechanisms for limiting their lifespan upon reaching a certain chronological age. Furthermore, it seems that these mechanisms can drive the evolution of yeast longevity towards maintaining a finite yeast lifespan within ecosystems. One could hypothesize that these mechanisms may involve the ability of the parental wild-type strain to secrete into growth medium certain compounds (small molecules and/or proteins) capable of inhibiting growth or even killing long-lived yeast mutants. Because these compounds may be responsible for the maintenance of a finite yeast lifespan within ecosystems, it would be important to identify them.
